# Rethinking One Health: Emergent human, animal and environmental assemblages

**DOI:** 10.1016/j.socscimed.2020.113093

**Published:** 2020-08

**Authors:** Alicia Davis, Jo Sharp

**Affiliations:** a Lecturer of Global Health, Institute of Health and Wellbeing, School of Social and Political Sciences, 27 Bute Gardens, Room 221, University of Glasgow, Glasgow, G12 8RS, Scotland, UK; b Geography & Sustainable Development, University of St Andrews, St Andrews, Fife, KY16 9AL, Scotland, UK

**Keywords:** One health, Global health, Zoonoses, East Africa, Assemblage

## Abstract

One Health perspectives are growing in influence in global health. One Health is presented as being inherently interdisciplinary and integrative, drawing together human, animal and environmental health into a single gaze. Closer inspection, however, reveals that this presentation of entanglement is dependent upon an apolitical understanding of three pre-existing separate conceptual spaces that are brought to a point of connection. Drawing on research with livestock keepers in northern Tanzania, in the context of the history of livestock policy in colonial and postcolonial East Africa, this demonstrates what an extended model of One Health - one that moves from bounded human, animal and environmental sectors to co-constitutive assemblages - can do to create a flexible space that is inclusive of the multiplicity of health.

## Highlights

• We show that One Health is based on conceptual separation
• Colonial and postcolonial health policy in East Africa enacted this separation
• Critical social science forces One Health to recognise interspecies entanglements.

A ‘laibon,’ or a spiritual leader and healer, provides guidance to people in Maasailand on issues ranging from settling disputes to gaining power to ill health. […] Having known a particular laibon informally for years, it was only on a trip to Tanzania in 2018, that I sought him out for a more formal interview as part of a study on zoonotic diseases (i.e. diseases transferrable from animals to people, including brucellosis and rift valley fever). I sat with him one late afternoon into evening as his sons and grandsons began to bring the animals back into the homestead for the day. As part of this study, we are re-thinking how human-animal-environmental interactions in rural communities influence disease, health, and wellbeing. Health challenges affect species/people in ways that are often difficult to express, they are layered and multi-dimensional, especially when it comes to livestock. Yes, these interactions are economic, as people rely on animals as either their primary or critical supplementary source of food or access to cash income (to purchase basic household needs). But it's also physical - as zoonotic diseases pose actual health risks to and have impacts on bodies (both human and animal). And, it's emotional - as people acutely feel the impact of their ability (or inability) to care for their families and their herds, as when they see those they care for suffering. It's also spiritual, as health and wellbeing are tied to belief in a higher power, for Maasai, Engai or God. As we sat there that evening, the Laibon explained what he saw when he looked out into his herds. (Davis, Fieldnotes, 2018)

## 1 Introduction

The excerpt that opens this paper is from an interaction with an old friend of one of the authors, conveyed in 2018 during fieldwork in northern Tanzania as part of a One Health (OH) project that sought to explore the drivers of zoonotic disease and livelihood change for livestock keepers. OH, an approach to health that recognises that human, animal and environmental health are systemically entwined, seems to have found its time and is increasingly taken up by both academics and policy-makers who are encouraged by its recognition of the interdependencies between human, animal and environmental health. While the concept is not new, and others (Woods and Bresalier, 2014; Woods et al., 2018) have highlighted the longer history of OH in other guises, Hinchliffe (2017: 160) has argued that this ‘unified and holistic approach to health’ emerged from a meeting of a US conservation agency in New York in 2004 (see also Friese and Nuyts, 2017; Hinchliffe, 2015; Brown and Nading, 2019). As health research is often critiqued for adopting a narrow focus only on the biomedical aspects of health (or, more accurately, of a specific disease), the OH approach should be welcomed. However, as it is currently applied, the concept, especially its form of “One World, One Health” (OWOH), is regarded by some social science commentators as only superficially covering social, political and economic processes, therefore reproducing a western-centric biomedical epistemology (see the special issue of *Social Sciences and Medicine* in 2015, edited by Craddock and Hinchliffe; see also Friese and Nuyts' 2017 review of the integration of posthumanism in public health/OH work, as well as Harrison's 2019 comparison of EcoHealth and OH frameworks).

In this paper, we want to develop these critiques, first to show how the apparent entangling of human, animal and environment in OH is actually based on deep conceptual separation, and second, to explore how this conceptual separation is replicated in the spatial politics of colonial and postcolonial management of the (health of) pastoralist populations of East Africa. While critical social scientists are attuned to the entanglements of human, animal and environmental health, this approach has yet to influence OH policy and practice. We will conclude with a proposal for an extended approach to OH. In this approach we regard health as an assemblage which recognises the always already entangled nature of people, other animals and the environment. Having worked on a number of interdisciplinary One Health projects we are all too aware of the “frictions” of such work (Craddock, 2015) which is often ensared by a biomedical-epidemiological focus. It is within these very collaborations and research that we have also seen a need to push the boundaries of OH, consider it as assemblage and incorporate posthumanist perspectives (Friese and Nuyts, 2017).

## 2 Modern(ist) One Health

OH in name and application appears to be an integrative and inclusive approach to solving increasingly complex global health challenges, recognising that in the majority of cases, human, animal, and environmental health are interconnected. For instance, the World Health Organisation (WHO, 2017) defines OH as:

an approach to designing and implementing programmes, policies, legislation and research in which multiple sectors communicate and work together to achieve better public health outcomes. […] Many of the same microbes infect animals and humans, as they share the eco-systems they live in. Efforts by just one sector cannot prevent or eliminate the problem. For instance, rabies in humans is effectively prevented only by targeting the animal source of the virus (for example, by vaccinating dogs).

There have indeed been very effective OH interventions, including the rabies example highlighted by the WHO (see Cleaveland et al., 2014), and some commentators have pointed to the *potential* for OH approaches to offer more equitable outcomes due to the broader scope of its understanding of disease contexts (for example, see Cleaveland et al., 2017). Yet, these primarily remain within the scope of epidemiological study.

However, there have been critiques of some forms of OH, particularly the tendency to universalise western health values (Craddock and Hinchliffe, 2015; Rock, 2017), put humans (and only some humans at that) at the top of a hierarchical structure of health (Brown and Nading, 2019; Hinchliffe, 2015), ignore the social and cultural contexts of health (Woldehanna and Zimicki, 2015), or ignore the political economies that often cause health disparities to begin with (Wallace et al., 2015). In the OWOH variant, Craddock and Hinchliffe (2015) see an approach that privileges western biomedical views of disease priorities, sitting within a tradition of western development approaches either disregarding “indigenous knowledges” or seeing them as superficial additions to business-as-usual (see Briggs and Sharp, 2004). OH is, they argue, fundamentally a western, modernist epistemology (Friese and Nuyts, 2017). Calls for more expansive OH approaches have suggested attentiveness to the social and political should be included (Woldehanna and Zimicki, 2015) not just as “context” but as places of friction and production (Craddock, 2015). We want to build upon these critiques in two ways, first to consider the ways in which OH conceptualises the relationships between human, animal and environment and, second, how it conceptualises health. In doing so, we hope to demonstrate the entanglements of humans, animals and other things as necessary to produce healthy outcomes.

**Fig. 1:**
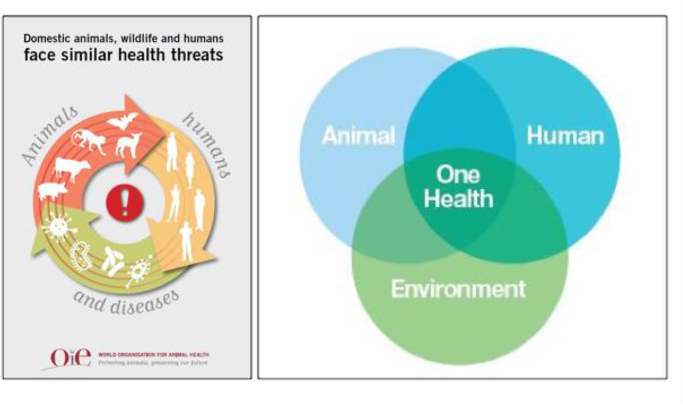
Examples of commonly used diagrams representing “One Health”. Left image by OIE: http://www.oie.int/en/for-the-media/onehealth/. Right image presented on WHO's Twitter feed: https://twitter.com/who/status/959023059737939968.

To address the first question, we need to consider the typical diagramming of OH. There are a number of different diagrams used (see Fig. 1 for two examples), but most illustrate intersecting circles or spaces of human, animal and environmental health to highlight shared interests and vulnerabilities. OH is presented as being inherently interdisciplinary and integrative: recognising entanglements and refusing narrow disciplinary focus. At first glance, then, this seems entirely appropriate. Yet closer inspection shows that this presentation of (what we view as an) entanglement is dependent upon three pre-existing separate conceptual spaces that can be brought to a point of connection (a conceptual separation that is reminiscent of Latour's (1993) famous critique of the modernist diagramming of culture and nature). Lezaun and Porter (2015: 100) explain that this separation is key to the ambition of One Health, which is “to contain pathogens by deploying devices that enclose humans and animals in specific, sterile, and segregated spaces.” They cite an FAO report on OH which illustrates this separation well through an emphasis on the “interface” between humans and other animals:

The interface between humans and animals is a critical juncture where zoonotic diseases emerge and re-emerge. This interface is continuously affected by increased globalization; the growth and movement of human and livestock populations … increased changes in ecosystems; changes in vector and reservoir ecology; land-use changes; and changes in patterns of hunting (FAO et al., 2011: 1).

The bringing together of three conceptual spaces to highlight intersections, then, actually reveals the foundational conceptualisation of bounded, separate and coherent identities. It is a move that parallels what Hinchliffe et al. (2016: 33) see as running through conceptualisations of threats to human life from infectious diseases:

They assume a dichotomy between a previously healthy inside and a pathogenic outside, with the crossing of the border between the two as a key moment of infection and disease. The resulting division of healthy bodies and disease bearing microbes presupposes a world of discrete and definable entities, with intact surfaces that may or may not come into contact.

In the context of OH, it is the borders—conceptual and material—between human, non-human and environmental objects which present the possibilities for similar transgressions. OH interventions are designed to police these borders to interrupt the transmission of disease but, notably, such biopolitical practices are ultimately enacted primarily to protect the life of humans. Nading (2013: 67) contends that it is in the very porosity of borders between the human and non human that biopolitics can arise and where the governmentality of health happens “through surveillance, medication, and regulation.” Thus, despite its integrated approach to thinking about health, OH is revealed to be built upon binary thinking which creates hierarchies and boundaries between humans and non-humans, beings and the environment, diseased and healthy bodies. These separations and hierarchies are deeply embedded in where the human (and, again, according to Hinchliffe (2015), *specific* humans at that) has oft been aligned as either ‘sacred’ or ‘pure’ and the non-human as ‘polluted’ or ‘profane’ (Douglas, 2003; Durkheim, 2005). Yet, in his work on western science, Latour (1993) has, famously, suggested that the separation of humans from nature is a fiction of modernity and that hybrids inevitably proliferate. In the case of human and animal health, Shukin (2009) suggests that the biomodality of the twenty-first century is “suggestive of a radical ontological breakdown of species distinction and distance under present conditions of global capitalism” (183). This points to an alignment of the “othering” of the non-human and the non-western. Shukin (2009: 186) claims that in the face of pandemic threat, “the sacrifice of potentially infectious (non-human) bodies so that others (humans) may live, […] simultaneously distinguish[es] racial ontologies in the global species body of humanity”. These are suggestive of the effects of global capitalism on bodies, separated by species yet increasingly linked by biosecurity concerns (Shukin, 2009). In Cairo in 2009, for instance, biosecurity discourse narrated the fear of a swine flu outbreak, leading to the (inhumane) killing of up to 300,000 pigs, in complete disregard for the ways these animals' lives were intertwined with the minority, marginalised Coptic Christian population. Both populations were represented as being dirty, diseased and threatening – despite the fact that not one human had tested positive for swine flu. The cull was presented as a small price to pay for the security of the national interest (see Tadros, 2010).

Borders run through the operationalising of OH, from the marginalisation of non-biomedical ways of knowing and being to the rendering of Others (racialized human and non-human) as “bare life”, as bodies that can be killed but not mourned, the dark side of biopolitical practice designed to support the life of wealthy, western, human bodies. It is a key point. The nature of OH diagrams imply an unproblematic aligning of interests of the three components, “a shared biological destiny,” as Wolf (2015: 6) has put it. It simplifies the interdependencies and entanglements between the human and non human (Rock and Degeling, 2015) though a focus on disease can sometimes bring these entanglements “into sharper relief” (Nading, 2013). OH therefore too easily ignores “more-than-human” solidarities, i.e. the ethical concern of assisting “non-human animals, plants, and places” as a matter of practice for public health (Rock and Degeling, 2015: 61). Thus, particular concerns about biosecurity and global pandemics that OH perspectives should help alleviate through more nuanced approaches (Mutsaers, 2015) may still fall short as currently conceptualised, especially if they rely on thinking in terms of ‘containment’, ‘risk’ exposure and reduction, or other forms of ‘control’ (see Woldehann and Zimicki, 2015) and continue to undervalue the “the ways animals are implicated in human health” (Brown and Nading, 2019: 9). Animals thus become critical actors of bio-*in*security; they are sites of transmission, contamination and are swept up in “controlled forms of surveillance”, further separating the world into “the virtuous and the pathological” (Hinchliffe, 2015: 34). Ignoring the relational spaces of human and non-human, reproduces biosecure capitalist production, “single truths” of “western triumphalism” (Law in Hinchliffe, 2015: 34). OH *could* offer a means to define and contextualize the interplay of human and non-human, and reveal their interconnections and interdependencies, much in the way that Tsing (2017) claims flexible communities and assemblages can be open-ended, precarious yet expansive.

This leads to the second aspect of our argument which focuses on the way in which OH understands the concept of health. Very little OH work focuses specifically on health; most is concerned with managing a specific disease or syndrome (such as swine flu or rabies). Thus, health is defined as an *absence:* an absence of disease or malady. Duff (2014: 65) contends that this exludes other possible understandings of health:

The idea that health may be defined in the negative – primarily in terms of the *failure to observe* a discrete set of ailments of conditions (Foucault, 1973: ix-xi) – neatly dispenses, of course, with the challenges of identifying the ends to which health may itself aspire.

While there has been some attention to how disease can bring human-animal entanglements and their intimacies into “sharper relief”, they often do so by taking the biomedical for granted (Nading, 2013: 60). There have been calls to “understand how humans contextualize their own health within animal and ecosystem health” (Lapinski et al., 2015: 54). Again, this is still often done wthin a biomedical framework, epidemiology, and with a focus on health related behaviours (Lapinski et al., 2015; Friese and Nuyts, 2017; Harrison et al., 2019). Thus, though the idea of health can be seen to be inextricably tied up with concepts of the social, political, cultural, economic and spiritual as well as the biomedical, in OH research, the focus is oft on disease, which tends to be understood explicitly as a biological matter. Some have drawn attention to the locus of disease and sickness as critically important in human/non-human entanglements (Nading, 2013; Rock, 2017), particularly as humans are “materially, economically, and even symbolically connected to animals” (Nading, 2013: 61 in Rock, 2016: 361), while others suggest that the political components of health and disease still remains underexplored (Hinchliffe et al., 2013). Looking at health as an entanglement and drawing from Deleuze and Guattari (1987), Nading (2013: 69) contends that human and non-human (i.e. animals, pathogens and spaces) become connected “in a process of mutual becoming.” Entanglements being non-linear, based on changing relations between the human and non-human (Ogden, 2011), help highlight the porosity of species borders, yet scientists often attempt to define these borders in order “protect life” (Ogden, 2011:2). A focus on diseases jumping in-between human/animal emphasizes yet another borderland enacted in these processes (Hinchliffe et al., 2013; Nading, 2013). And as Duff (2014: 175, drawing on the work of Canguilhem (2012)) explains, whenever “health is first conceived as the absence of disease, the temptation to convert health into a measure of the body's ‘natural’ biological order inevitably appears”, hence removing attention from non-biomedical causes and contexts.

This shift to the biomedical has implications for the ways in which agency is imagined. Paul Farmer (2001: 258) insists that exaggeration of patient agency “is particularly marked in the biomedical literature, in part because of medicine's celebrated focus on individual patients, which inevitably desocializes”. For him, disease is the outcome of social difference rather than biology (see also Wolf, 2015: 6). As Farmer (2001: 79) has explained, “sickness is a result of structural violence: neither culture nor pure individual will is at fault; rather, historically given (and often economically driven) processes and forces conspire to constrain individual agency”. This approach leads to the stigmatisation of particular people, as it ignores the role of structural violences that render some bodies more vulnerable to diseases than others; that make it possible for some agents to act on the “good” knowledge they have but forces others to continue with risky practice. Where Nading (2013) contends that life, rather than being a baseline from which culture and society spring, is best understood as ongoing, dynamic and made up of material and symbolic relationships among humans, other lifeforms, and their environments, Hinchliffe (2015) calls for extended social science approaches that allow for multiple logics, knowledges, and practices to understand what makes health. “Clearly, health is a multi-species matter” (Hinchliffe, 2017: 172).

Hinchliffe (2017: 163–4), following Law and Mol (2008), also sees the “one worldism” of certain OH approaches as drawing on particular assumptions about the world: it assumes the world is comprised of separate surfaces, volumes and collisions, it assumes a naturalised epistemology where the world is rendered legible to the viewer, and it claims to be universal but is instead embedded within western practice. We will now turn to the practices that have remade the African landscape through what could be seen as an uncritical OH epistemology. In health policy, African landscapes have become a site of health transgressions, border creations, and entanglements of health. By paying critical attention to the entanglement of human and animal, to their mutual becoming and “shared suffering” in the context of capitalism and post-colonial encounters, social scientists can productively destabilize the anthropocentrism of conventional public health. We explore tensions in the management of livestock in Northern Tanzania between the colonial and postcolonial governmentality of modern veterinary policy and the more entangled worldview and practice of livestock keepers themselves.

## 3 Creating healthy African landscapes

European colonialism sought to bring enlightenment to the “Dark Continent”. European colonisers regarded the lack of visible transformation of the African landscape by the native population as evidence of their lack of civilisation, and as evidence that they should be considered part of nature rather than culture in colonial taxonomies (Adas, 1989). Colonial ideals of what an African landscape *should* look like placed landscapes in binary terms; those of “wilderness” and those of “domestication”, with many places deemed “degraded” or as spaces of “unfulfilled potential” when occupied by Africans (Atieno-Odhiambo and Cohen, 1989; Shetler, 2007; Davis, 2010). Thus, colonial governments, settlers, and eventually independent states complied with these constructs of use, misuse, and degradation of Africa. For example, areas that lacked cultivation were seen as ‘unused’ or their productivity not yet ‘improved’ by the indigenous population and were moralized through a lens that regarded cultivation “as part of the extension of the Lord's kingdom” (Hodge, 2007: 25). Landscapes were thus either viewed (and treated) as ecological (and set aside for conservation purposes) or moral (and targeted for improved, ‘proper’ productive use, i.e. agricultural development). Within this typology, open, undeveloped rangelands full of wildlife were categorized as wild natural landscapes or neutral spaces. In contrast, farming, which brought the land into productive use, was regarded as the basis for a right of ownership.

The making of the African landscape is also linked to the history of European abolitionism and Christian proselytizing and crusading; the rise of capitalism; industrialisation and destruction of European natural resources; the rise of natural sciences and new conceptualizations of nature, wilderness, and protection; and new sciences of clinical pathology and empirical investigations of bodily interiors (Comaroff, 1991; Comaroff and Comaroff, 1991). Much has been written about European colonists' frustration at the apparent refusal of the colonial landscape to fit into their categorisations and idealized imaginary: “For many postcolonial governments, this ability to rearrange the natural and social environment became a means to demonstrate the strength of the modern state as a techno-economic power” (see: Mitchell, 2002: 21; Adas, 1989; Briggs and Sharp, 2009). The modern state here is epitomised as achieving the transformation and organisation of nature and society's productive capacity, originally set down by the colonial powers.

The wildness and mysteriousness of East and Southern Africa has been embedded in the mythos of the “dark continent”, to be simultaneously preserved and defended; tamed and dominated; *and* made “productive”. The contrasts between an “Eden” and the “dark continent” were not incompatible. Colonial rules, laws, and governance served both ideals so long as resource control was removed from local populations and control granted to various “experts” (Neumann, 1998). Values and meanings of resources were reshaped (Williams and Williams, 1977) and life itself (wild, domestic, human, animal, land) subsumed under colonial control (with the help of science, expertise, and subsequent hierarchies of control). Knowledge of and control over organic life was a mission of colonial and European science, and it affected not only geographies but bodies as well (Comaroff, 1991).

## 4 Disease control and the colonial state

The idea of controlling disease, particularly livestock disease, in East Africa needs to be set within this historical context. Colonial governments embedded structures and ideologies of control into health systems (both for livestock and human health) which were then adopted by independent states. These structures reified “assumptions, tensions, and contradictions latent in the colonial state” through constructions of disease (Waller, 2004: 46). Waller (2004: 46) contends that these assumptions act “as a lens through which to examine fissures in state and community” as well as understanding how knowledge, power, and imposition of solutions “pitted” the state against “established African pastoral practices”. Historically then, “weapons against disease included legislation, boundaries, fences and policemen, as well as the microscope and the needle” (Waller, 2004: 46). Boundaries were not only part of the battle against livestock disease but part of a justification for control over colonial subjects, particularly those considered to be ‘unruly’, ‘irrational’, and ‘fierce’, like pastoralists. These efforts established change that both undermined and solidified pastoralist identity itself, for example, amongst the Maasai in southern Kenya and northern Tanzania. Pastoralist identities became more spatially bound while the networks of interaction, reciprocity, and exchange were monitored, fissured, and reshaped (Hodgson and Schroeder, 2002; Hodgson, 1999, 2001). This framing was made possible, in large part, because of what Hodge (2007: 25) notes as the characterisation of the pastoralist as bodies “roaming over” the landscape rather than being “proper” inhabitants, which has persisted in land policies in much of Africa. African bodies had long been demonized, explored, destroyed, and controlled by colonial powers (Comaroff, 1991). Medical and scientific research was used to control slaves, to justify European racial superiority, and to control populations within and outwith colonies (Comaroff, 1991*)*.

Disease control for Maasai in Kenya, for example, had two fronts: those targeting the indigenous/subsistence and those that were market oriented (Waller, 2004, 2012). In Tanzania as well, systems of taxation, isolation, bordering, monetization, and commodification of livestock based livelihoods were key components of the colonial state, meant to dismantle pastoralism (Hodgson, 1999). These systems of control were based on misconceptions that pastoralism was a historical remnant “conservative, specialized, and unchanged … until the great pandemics and the establishment of colonial rule” (Waller, 2004: 47). A key component of control and separation of the human and animal occurred through the division of veterinary and agricultural services, which contended that veterinarians were the ‘experts’ meant to handle animal disease matters only, while agricultural specialists dealt with other farm based livelihoods. As this biopolitical regime developed greater specialisation, further separations occurred between dairy producers, meat producers, subsistence producers and market based producers, each with their own distinct office/sector to control, tax, and oversee (Waller, 2004, 2012). Veterinary services focused on disease demanded compulsory procedures such as “fencing, dipping, immunization, removal of squatters”, as it was “disease itself” which was “central to transforming the economic and social environment” of East African rangelands (Waller, 2004: 67). The state was able to use quarantines, separations, and boundaries to distinguish “clean and dirty” space, those that were “protected spaces in the landscape”, and it was western veterinary knowledge that was privileged over pastoralist knowledge in controlling and understanding disease (Waller, 2004: 80). Imposed quarantines by the colonial state were “blunt instruments” used to separate European herds from African herds (Hodgson, 1999) and which identified and punished those with ‘diseased’ animals, clearly distinguished from the healthy.^1^ Finally, boundaries and taxes were not just enacted for the separation of healthy/unhealthy animals and people, but also to control the supposed impacts of pastoralists on the environment. Improving stock and shifting to market based livelihoods was thought by the colonial regime to bring the additional benefits of preventing soil erosion, environmental degradation, and other detrimental effects of pastoralism on the rangelands. These conflicting interests and boundary drawing practices still remain today (Hodgson, 1999).

Pastoralists, meanwhile, had long practiced what can be called experience-based treatment of disease (Waller and Homewood, 1997), which is embedded in their own spiritual beliefs and relations to nature. Disease was seen to be part of the environment and part of the landscape of pastoralism. Maasai had methods of disease control that were management based. For example, ticks were controlled by burning grasses, while East Coast Fever (ECF) and other diseases like rinderpest built immunity in herds through exposure or the constant “circulation” of disease through herds, maintaining immunity levels and reducing the impact of epizootics (Waller and Homewood, 1997). Thus, during the colonial period there emerged two types of pastoralism: the white settler and the native, “divided in how they saw disease and in how they responded to it” (Waller, 2004: 49). Settlers depended on the state for protection, which relied on scientific approaches to livestock management, whereas “Africans looked to themselves and relied on experience” (Waller, 2004: 49). These dichotomous and opposing views, expressed even by social scientists, ignored the realities and hybridities of pastoralist approaches to their animals and livestock management as discussed below.

Yet the entanglements between colonial governance and the use of language of disease is clear:

Disease was simultaneously a real threat and a useful metaphor. It's presence in endemic form in African herds beyond the boundary not only justified separation but also provided a way of visualizing the contrast between settlement and savagery, progress and stagnation (Waller, 2004: 51).

The movement of pastoralists with their livestock across space threatened these solidified boundaries, dichotomies, and criminalized “customary pastoral practice” (Waller and Homewood, 1997: 51). The colonial regime played up fears of the unruly black African bodies so as to emphasize regulation and stop (illicit) movement through branding, counting, and registration (in Kenya). Similarly in Tanzania, the desire to monetize, quantify, and commoditize played into efforts of separation and dichotomization. ‘Discovery’ of diseases like East Coast Fever (ECF) led to ‘cleansing’ pastures to get rid of the tick vectors, revealing the material (cleansing made herds more vulnerable to reinfection) and political effects of the colonial geographical imagination:

[to] the triumphant march of colonial science, ECF symbolized the African environment at its most intractable, and measures against it displayed the processes of demarcation and control through which the colonial state made itself. In a sense, ticks, especially in the enclaves of European order, were the insect equivalent of the unruly Africans whose ‘wandering’ herds gave them passage (Waller, 2004: 55).

Veterinary policies were closely linked to state policies supporting land tenure changes that provided legal means for “consolidating and isolating Maasai and their herds in a distinct bounded area and restricting their movement and interactions outside of the area” (Hodgson, 2001: 80). Veterinary officers were less concerned with helping Maasai than with protecting the land, livestock, and livelihoods of European settlers, and to some extent, other Africans, “from the ‘dangers’ of Maasai interference and entanglements” (Hodgson, 2001: 80) such as disease or ‘lawless’ behaviour. The use of taxation, boundary making, and dichotomization did not end after independence in either Kenya or Tanzania. These methods ignored the lived and entangled realities of pastoralists. For the Maasai and other pastoralists in the region, state-centred livestock policies further embedded livestock and human health policies, processes and provisioning of services in separation and bordering.

## 5 Disease, livestock and health in East Africa

Again, the Laibon's story reveals the impacts of (post)colonial governance:

At a recent visit, to just say hello the Laibon's eldest son, Sirongoi [all names changed] began to recount a recent district government meeting he'd been at as a local representative. At the meeting, a new ‘branding’ program by the national government was discussed. Sirongoi explained the publicly stated intention of the program and then what he saw as being the “real” intention. The government says they want to be able to identify animals from various districts, to track the livestock trade, keep up with cattle movements (in part for disease surveillance) and to cut down on cattle thievery, and, “‘protect’ cattle keepers”. But the group of Maasai elders on that Sunday all agreed, there was something else afoot. Maasai, the Laibon explained, already have ‘brands’. “This clan has a brand, a family has a brand,” to mark who owns which animals. “So” he said, “if Laibon's animals are by the water, and Sirangoi's arrive, you can say ‘oh, those are Laibon's, those are Sirangoi's and they can be separated if they mix, or you can admire the way a certain man keeps his cattle in good health.” “I ask you,” the Laibon continued, “if the government puts their brand on the animals, who does it show owns them? We think that's a sign that they will then own them. … He went on, “since I was a young boy, the government has tried to take Maasai cattle, prevent us from grazing in our own lands, reduce our numbers. Always they think we are destroying, when that is their intent.” Sirongoi added, “its not even so much the brand, what's a brand? A mark, fine you mark my cattle … but they also come with a paper, to ask, how many of this, how many of that do you have? What is the purpose of this? … Just say, you want to tax them, to confiscate the animals of people who don't comply, tell us so we know how to proceed. (Davis, Fieldnotes, 2018)

In Maasai pastoralist epistemologies, there is no clear line of separation between human and animal, animal life and the surrounding environment. Maasai derive not only their livelihoods from livestock, but their ‘traditional’ belief systems, stories, songs, and everyday ways of being have integrated their knowledge of and relationships to their animals (Talle, 2004; Spear and Waller, 1993; Galaty, 1982, 1983; Hodgson, 1999, 2001; Floyd, 2017). Historically, livestock formed the basis of the economic modes of production, social connections within and outside of Maasai communities, spiritual connections to God and the landscape, and physical nourishment (these entanglements conjour Evans-Pritchard's (1951) description of the Nuer who saw that the “social idiom is a bovine idiom” (p. 19)). Thus Maasai already experience and embody “more-than-human solidarities” that OH strives for. While traditional means of production and connection to their livestock still underpin much of Maasai life, there exists a hybridity between what some may deem ‘modern’ and ‘traditional’ (as discussed above in terms of health systems) which is infused into Maasai beliefs (e.g. long standing influx of Christianity), livelihood practices (livelihood diversification to day labour, farming, mining, tourism enterprises, livestock trading, professional white collar work), livestock management (use of fodder, sedentarism, improved breeds, market production), governance (civil society participation, state laws and regulations, changing internal dynamics) and health (biomedical human and veterinary services, medicines, practices). When caring for their livestock, Maasai have created experience-based and “pluralistic” knowledges that incorporate biomedicine and veterinary knowledge into everyday practice. Beinart and Brown (2013) contend that this pluralism is consistent with other forms of health knowledges where there is not a clear demarcation or dichotomization of ‘African indigenous knowledge’ versus ‘scientific’ reality (see also Briggs and Sharp, 2004). The health of animals is wrapped up in hybridity, pluralism, and bricolage, but the different knowledges are not considered equal (Beinart and Brown, 2013), particularly by current state management structures.

In contemporary Tanzania, livestock, land use, and veterinary policies uphold colonial ideas about the negative effects of pastoralism on the landscape, for people, and for control of disease. National policies set a tone for what kinds of livestock-keeping the state is interested in. For example, the National Livestock Policy (URT, 2006) establishes support for “modernization” and “industrialisation” of the livestock “sector”, particularly through privatization. Thus policy also is decidedly pro-privatization or recommends private-public partnerships to support livestock markets, health services, and disease control. Yet the policy also acknowledges that the private sphere is often “weak” or “inadequate” to meet the needs of livestock keepers. The policy simultaneously denigrates non ‘official’ forms of knowledge and expertise that exist outside of recognized private spheres. Pastoralists and their “Indigenous Technical Knowledge” [sic] are also disparaged as deficient (URT, 2006: 35–36).

Likewise, communal land tenure, upon which pastoralism depends, is presented as constrained by lack of knowledge about privatization, legal ownership, and “proper land utilization for sustainable livestock production and productivity” (URT, 2006*)*. This lack of tenure security (which, according to policy, should be remedied through privatization and individual land ownership) is blamed as a source of “social conflict between livestock farmers and other land users, land degradation and spread of animal diseases” (Rule 3.23.1). The Livestock Policy claims that communal grazing encourages, “free and uncontrolled movements” of people and animals (Rule 3.7). These unrestrained movements cause “overgrazing, degradation of the environment” (Rule 3.5.5) and “overstocking” (Rule 3.7). Furthermore, the policy ties overstocking directly to, “social and cultural perception of some livestock farmers … for prestige and security” as well as these uncontrolled movements. The policy insists that these practices are not “proper livestock stocking” nor do they represent “good husbandry practices” (Rule 3.7).

Maasai in Tanzania and Kenya have historically practiced transhumant pastoralism (seasonal mobility) that ‘ignored’ the borders conceived and erected by states (first colonial and then post-colonial). Boundaries did exist between other groups of pastoralists, farmers, and even Maasai of different sections or clans. Livestock mobility was driven by the search for fodder or water, or the escape of disease or conflict. Historically herders moved at their own risk (such as meeting hostile neighbours; encountering diseases through proximity to wildlife or other herds; or failing to find adequate grasses). Communally governed rangelands and strong practices of reciprocity for water and pasture mitigated some of these potential risks. However, as geopolitical borders of the state have expanded through erecting protected areas (to preserve ecological health) (Galvin, 2008), wildlife corridors (Goldman, 2009), or village boundaries (Goldman et al., 2016; UCRT, 2010), new risks came with mobility—animals could be confiscated and fines enacted on pastoralists as control mechanisms of pastoral lives. New rules (as recent as 2017 and 2018) are increasing fines, confiscations, and sell-offs of animals for those accused of “smuggling” livestock across borders (Tairo, 2018). In other words, the once cross “border” movements of livestock are now marked as illegal.

Additionally, increased privatization of land in Tanzania means that open rangelands are decreasing in size, have restrictions of access, and consequently fines and conflict are associated with transgressing borders. Maintaining common grazing land and healthy herds is increasingly complicated and challenging as villages divide further (with increased population or from conflicting uses), land parcels become smaller, land use competition increases, and drought (which causes significant movements from local areas) becomes more frequent (Behnke, 2018; Galvin, 2009; Hobbs et al., 2008; Leeson and Harris, 2018). Paralleling these trends, the boundaries between different life forms (human and non-human) have also shifted. As pastoralist livelihoods necessarily diversify, particularly in terms of engagement in agriculture, increased livestock sales, livestock market diversification and market-based movements (within and across state boundaries), there are further boundaries between people and their livestock. By embedding these relationships in commodification and capital, taxation, and individual ownership, livestock health becomes further entrenched in state politics and goals.

In 2017–18 a new program was introduced to address overstocking and unrestricted movements. It was to be enacted through the introduction of government ‘branding’, as mentioned in the Laibon's story above. A new mechanism of control, this program was piloted in northern Tanzania as an attempt to brand animals at village locations, so that district officials could identify animals moved out of their areas of origin. In marking these animals, movements could be controlled or restricted (and perhaps taxed). To the Laibon, this policy clearly builds on the historical experience of governance which has sought to enforce boundaries to control the bodies of the pastoralists and their animals in the service of controlling pathogens. This explains the Laibon's wariness of the new regulations, and the likely non-compliance of many Maasai with rules that are perceived to be part of an on-going attack on Maasai life.

## 6 Conclusion: Towards an extended One Health

A spiritual component of health resides on several planes for Maasai, where healing is impacted by faith, belief, and prayer. Because health is also intimately tied to livestock and their health, whom Engai created, gifted and entrusted to people, Maasai are responsible for these animals. Thus when animals (and grasses) are unwell, people are unwell. The Laibon spoke about the connection he feels to his animals, and what he thinks/feels when they are unwell. He explained why he chooses some animals over others to sell, to slaughter, to vaccinate, to treat, to keep until they are old are barely passable for food or other useful ‘economic’ purpose. We talked about how, from a young age, children become attached to particular animals. Their character, color, demeanor, their usefulness, their personalities are all tied into this as are Maasai origins and clan affiliations. The Laibon explained how he hates to see his animals suffer with disease or from drought conditions because it not only impacts his children and grandchildren but also hurts his relationship to God when they are unwell. (Davis, Fieldnotes, 2018)

Blue and Rock (2011) argue that zoonotic disease specifically “engender[s] trans-biopolitics” because it causes us to “assess the relative value of human and nonhuman animal bodies, and that prioritizes the vitality of some species while at the same time marginalizing others” (Porter, 2016: 148). This is variously achieved through market movement regulations, livestock ‘modernization’ and censuses. Biopolitics are expanded into this environment through the making of subjects (human and animal) who need surveillance, ‘control’ and boundaries to contain zoonoses or to contain rangeland degradation. This is manifest in the Maasai example, and for pastoralists more generally. It is not just limited to the relative valuing of human/animal health issues, but occurs through differential valuing of animals (colonist/pastoral, commercial/pastoral) and of the health of certain environments too. The “depths, intensities, and affective complexities of social relations between humans and animals” (Brown and Nading, 2019: 5) is often ignored by policymakers, the state, researchers in health, and health practitioners. These relationships are not reflective of “sentimentality of mutuality and entanglement”, but demonstrate what Brown and Nading (2019: 5–6) might suggest exemplifies what happens when “multispecies well-being is enabled (and sometimes harmed) across intimate, institutional, and governmental scales”. This runs through OH from the marginalisation of non-biomedical ways of knowing and being to the rendering of Others (racialized human and non-human) as ‘bare life’, as bodies that can be killed but not mourned, the dark side of biopolitical practice designed to support the life of wealthy, western, human bodies. This is a key point. The nature of OH diagrams imply an unproblematic aligning of interests of the three components, “a shared biological destiny,” as Wolf (2015: 6) has put it. Calls to integrate OH platforms into national and international health agendas (Smith et al., 2015) are then, not enough. The historical narrative we have presented here, however, reveals the power relations, tensions and contradictions emergent from an attempt to impose a Western OH model onto the East African landscape, a landscape that is illustrative of entanglement.

Reimagining OH as an entanglement, rather than a digram of overlapping spaces, allows for a recognition of connections and transformation through the emergent properties of an assemblage of human, animal, and other things. Assemblage is a much-defined term, but we follow Tsing's (2015: 22) definition:

Ecologists turned to assemblages to get around the sometimes fixed and bounded connotations of ecological ‘community.’ The question of how the varied species in a species assemblage influence each other – if at all – is never settled: some thwart (or eat) each other; others work together to make life possible; still others just happen to find themselves in the same place. Assemblages are open-ended gatherings. They allow us to ask about communal effects without assuming them.

This approach does not deny the impacts of zoonotic diseases on human and animal populations, nor does it ignore the impacts of the power to legislate, control or eat others in the assemblage; it does reveal the effects of the imposition of Euclidean spaces of biopolitics in the conceptual separation of species and in the governance of people and their animals. Hinchliffe et al. (2016: xiv) use the term “pathogenicity” to “highlight that infectious disease is always more than a matter for pathogens alone”; the same is true for zoonotic disease, and the wider understanding of health among human and animal populations. Similarly, healthy animals, humans, and environs for Maasai are then tied to assemblages of bodies, objects, and spaces created, co-created, and which “afford each other their existence and their capabilities” (Mol, 2010: 265). Mol's conception that the body is multiple means that “there is more than one way for disease to take place” (Greenhough, 2011: 135) but as importantly, more than one way to seek health. Thus, this is more than a conceptual point. OH interventions that focus on a singular disease through an epidemiological lens miss the ways in which people are embedded within multiple assemblages, and thus, may not work due to a failure to understand the entanglement of health in aspects of life beyond the biomedical. OH, as an integrative, multi-disciplinary approach, offers the *possibility* of providing such a space for multiple voices, but only if it is extended to consider health in all of its forms, rather than focusing narrowly on the absence of disease. Maasai themselves recognise a fluidity to their borders, to their herds, to identity, to health, whereas the state has continued to erect more or solidify existing borders.

The conceptual division between humans, other animals and the wider environment played out in the history of health management in East Africa clearly parallels the diagrams of OH and the continual focus on disease specific concerns and containments. It stands in stark contrast to the understanding of health outlined above by the Laibon and critical social science engagements with OH. For the Laibon, there are no separable human, animal and environmental spaces to be brought together because, for him, they are inseparable to start with. Thus when he implored us to understand, “when my animals are unwell, I am unwell” he was revealing the complexity of lifeways, assemblages, OH, and beyond. We take the Laibon's explanation of health as an inspiration for an extended model of OH that embraces their entanglements rather than separation, one that shines a light into the liminal spaces and one that recognises how and where borders are enacted, how history can be recreated, and where power connects and diffuses (Haraway, 1999). This paper highlights the need for One Health projects to engage with critical social scientists who can work with communities to ensure other perspectives are taken seriously in the conceptualisation of health “problems” and attempts to co-produce better health outcomes.

## Author statement

Alicia Davis and Jo Sharp share joint credit on this paper including on conceptualisation, methodology, analysis, data curation, writing and review. Ethnographic data collection was conducted by Alicia Davis.

## Declaration of competing interest

None.

## Footnotes

1 For example, this occurred with widespread quarantines of animals with contagious bovine pleuropneumonia (CBPP), disallowing indigenous breeds and herds from entering the market economy.
